# Lipoic Acid Synergizes with Antineoplastic Drugs in Colorectal Cancer by Targeting p53 for Proteasomal Degradation

**DOI:** 10.3390/cells8080794

**Published:** 2019-07-30

**Authors:** Carina Neitzel, Nina Seiwert, Anja Göder, Erika Diehl, Carina Weber, Georg Nagel, Svenja Stroh, Birgit Rasenberger, Markus Christmann, Jörg Fahrer

**Affiliations:** 1 Institute of Toxicology, University Medical Center Mainz, 55131 Mainz, Germany; 2 Rudolf Buchheim Institute of Pharmacology, Justus Liebig University Giessen, 35392 Giessen, Germany; 3 Division of Food Chemistry and Toxicology, Department of Chemistry, Technical University of Kaiserslautern, 67663 Kaiserslautern, Germany

**Keywords:** lipoic acid, p53, ubiquitin, proteasome, mitochondria, anticancer drugs

## Abstract

Lipoic acid (LA) is a redox-active disulphide compound, which functions as a pivotal co-factor for mitochondrial oxidative decarboxylation. LA and chemical derivatives were shown to target mitochondria in cancer cells with altered energy metabolism, thereby inducing cell death. In this study, the impact of LA on the tumor suppressor protein p53 was analyzed in various colorectal cancer (CRC) cell lines, with a focus on the mechanisms driving p53 degradation. First, LA was demonstrated to trigger the depletion of both wildtype and mutant p53 protein in all CRC cells tested without influencing its gene expression and preceded LA-triggered cytotoxicity. Depletion of p53 coincided with a moderate, LA-dependent ROS production, but was not rescued by antioxidant treatment. LA induced the autophagy receptor p62 and differentially modulated autophagosome formation in CRC cells. However, p53 degradation was not mediated via autophagy as shown by chemical inhibition and genetic abrogation of autophagy. LA treatment also stabilized and activated the transcription factor Nrf2 in CRC cells, which was however dispensable for p53 degradation. Mechanistically, p53 was found to be readily ubiquitinylated and degraded by the proteasomal machinery following LA treatment, which did not involve the E3 ubiquitin ligase MDM2. Intriguingly, the combination of LA and anticancer drugs (doxorubicin, 5-fluorouracil) attenuated p53-mediated stabilization of p21 and resulted in synergistic killing in CRC cells in a p53-dependant manner.

## 1. Introduction

The natural compound α-lipoic acid (LA) is a chiral fatty acid harboring a disulphide bond, which can be reduced to dihydrolipoic acid (DHLA) [1]. The biologically active (*R*)-enantiomer represents an essential co-factor in mitochondrial multi-enzyme complexes performing oxidative decarboxylation (alpha-ketoglutarate dehydrogenase and pyruvate dehydrogenase). Thus, it represents a crucial player in the citric acid cycle and aerobic metabolism. In addition, LA and DHLA form a potent redox couple displaying remarkable anti-oxidative properties—e.g., by chelating divalent metal ions and by regenerating vitamin C [2,3,4]. LA was further shown to act anti-inflammatory by downregulating the pro-inflammatory NF-κB pathway [3].

LA is not only de novo synthesized endogenously in mitochondria, but also occurs in animal sources such as meat and in vegetables, in which it is most often covalently bound and therefore negligible for dietary uptake [5,6,7]. Marketed as antioxidant, LA is available as food supplement, and drug approval has been granted for treatments of chronic diseases associated with high levels of oxidative stress, such as diabetic polyneuropathy [8]. 

Recently, studies have investigated the potential of LA and its derivative CPI-613 as candidate drugs in the treatment of various types of cancer in vitro and in vivo [9]. Regarding LA, cytotoxic and tumor growth-inhibiting effects against a panel of different cancer cell lines have been demonstrated (colorectal, lung, breast, thyroid, skin) [10,11,12,13,14]. Treatment with LA generally induces imbalance of reactive oxygen species (ROS) release with concomitant loss of mitochondrial membrane potential culminating predominantly in apoptotic cell death via the intrinsic mitochondrial pathway [15,16,17,18], in which p53 was shown to be dispensable [15]. Furthermore, the autophagic machinery is triggered upon treatment with LA as a means of cell survival [13].

Previous studies indicated a putative synergistic action of LA and chemotherapeutic agents—e.g., 5-fluorouracil (5-FU) or etoposide—although the underlying mechanism remains to be elucidated since its multifaceted mode of action targets a plethora of cancer hallmarks [9,15,19]. In addition, LA was shown to target *O*^6^-methylguanine-DNA methyltransferase (MGMT), which is involved in the repair of alkylation DNA damage [20], for its degradation and thereby increases the cytotoxic effects of the alkylating anticancer drug temozolomide [13].

In healthy tissue, the tumor suppressor protein p53 is an important regulator of the DNA damage response (DDR), cell cycle progression, and apoptotic cell death [21]. Upon DNA damage, p53 is able to activate the expression of DNA repair genes—e.g., *GADD45A*, *XPC*, or *MSH2* [22]—intervene in the cell cycle via *p21* upregulation or causes transcription of pro-apoptotic genes such as *BAX*, *PUMA*, or *NOXA* [23,24]. The p53 protein is tightly controlled by post-translational modifications such as ubiquitination and phosphorylation [25], and is further modulated by the cellular redox state [26]. Mutations of p53 in cancer cells lead to either inactivation (loss of function) or hyperactivation (gain of function), both of which are crucial alterations resulting in an abrogation of its tumor suppressive functionality [27,28]. Colorectal cancer (CRC) is the third most frequently diagnosed cancer worldwide and 5-year-survival-rates are still devastating, stressing the need for improved therapy approaches [28]. Interestingly, approximately 50% of all colorectal tumors bear p53 mutations, prevailing in distal and rectal tumors [28,29]. Previous studies in different cancer cell lines indicated a differential p53 expression level upon LA treatment. On the one hand, depletion of p53 following LA treatment was observed [30], while on the other hand phosphorylation of p53 without changes of the total p53 protein level [31,32] or even a stabilization of p53 [19] were reported.

Triggered by our observations that p53 is dispensable for LA-induced cytotoxicity in CRC cells and that LA induces degradation of the redox-sensitive MGMT protein, we aimed to shed light on the effects of LA on p53 in CRC. At first, we studied the impact of LA on p53 on protein and mRNA level in various CRC cell lines and assessed the p53 transcriptional response. Subsequently, the generation of ROS by LA and the influence of anti-oxidant supplementation on p53 depletion was evaluated. Next, the involvement of different pathways such as autophagy and the proteasomal degradation machinery as well as post-translational modifications were analyzed, making use of different pharmacological inhibitors and genetic means. Finally, we set out to evaluate putative synergistic effects of combining LA and antineoplastic drugs used in CRC and other malignancies. 

## 2. Materials and Methods

### 2.1. Material

R(+)-LA, chloroquine (CQ), *N*-Acetyl-Cysteine (NAC), and MG132 were purchased from Sigma (Deisenhofen, Germany). The anticancer drugs doxorubicin (Doxo) and 5-flurouracil (5-FU) were from Medac (Wedel, Germany) and provided by the pharmacy of the UMC Mainz. The Nrf2 inhibitor ML385 and curcumin were obtained from Hycultec GmbH (Beutelsbach, Germany) and MDM2 inhibitor Nutlin-3a was from Selleck Chemicals (Houston, TX, USA). 

Primary antibodies included Hsp90α/β (F8, mouse monoclonal; Santa Cruz, no. sc-13119), p53 (DO-1, mouse monoclonal; Santa Cruz, no. sc-126), p53 (FL-393; rabbit polyclonal; Santa Cruz, no. sc-6243), p62 (mouse monoclonal; Santa Cruz, no. sc-28359), LC3B (rabbit monoclonal; Cell Signaling Technology, no. 3868), ATG5 (rabbit monoclonal, Cell Signaling Technology, no. 12994), ubiquitin (mouse monoclonal; Santa Cruz, no. sc-8017), Nrf2 antibody (rabbit monoclonal; GeneTex, no. GTX103322), MDM2 (mouse monoclonal; Santa Cruz, no. sc-56154), heme oxygenase-1 (HO-1; rabbit polyclonal; GeneTex, no. GTX101147), as well as p21 (C-19, rabbit polyclonal; Santa Cruz, no. sc-397). Monoclonal PARP-1 antibody was provided by Dr. Alexander Bürkle (University of Konstanz, Germany). Secondary antibodies conjugated with horseradish-peroxidase were purchased from Santa Cruz (anti-mouse) and Cell Signaling (anti-rabbit).

### 2.2. Cell Culture and Treatments

The human CRC cell line HCT116 and isogenic p53-deficient HCT116 cells were generously provided by Dr. Bert Vogelstein (John Hopkins University, Baltimore, USA). LS174 cells were a kind gift of Dr, Thomas Brunner (University of Konstanz, Konstanz, Germany). SW48, HT29, and RKO cells were provided by the Institute of Toxicology, University Medical Center Mainz. HCT116 and RKO were grown in DMEM, LS174T in IMDM, and HT29 as well as SW48 in RPMI1640 medium supplemented with 10% fetal calf serum and 1% penicillin/streptomycin at 37 °C in humidified atmosphere of 5% CO_2_. Media and supplements were obtained from Gibco Life Technologies (Darmstadt, Germany) or PanBiotech (Aidenbach, Germany). All cell lines were mycoplasma negative.

LA was prepared as a 200 mM stock solution in 100% ethanol and added to the cell culture medium as indicated. Ethanol served as solvent control (0 µM LA). NAC was directly dissolved in cell culture medium (5 mM). In combination studies, cells were pre-incubated with 5 mM NAC for 2 h prior to LA treatment. When CQ (100 mM in H_2_O) was used to block autophagy, CQ was added 16 h prior to harvesting of the cells in a final concentration of 20 µM. In experiments using MG132 (13 mM dissolved in DMSO), the proteasome-inhibitor was added 16 h after LA treatment using a dose of 10 µM. ML385 and Nutlin-3a were dissolved in DMSO and used at a final concentration of 5 µM. Inhibitors were added to cell culture medium 2 h prior and 24 h after LA-treatment.

Combination treatments with anticancer drugs (Doxo/5-FU) included 44 h of incubation with LA plus 4 h of treatment with Doxo/5-FU for western blot analysis and 48 h of incubation with LA plus 72 h of treatment with Doxo/5-FU for ATP assays and Annexin V/PI stainings.

### 2.3. Preparation of Protein Lysates and Cell Fractionation

Whole-cell extracts and cell fractionation was performed as described [13]. After indicated time points, cells were harvested and whole cell lysis was performed. In short, cells were lyzed in buffer containing 25 mM Tris-HCl pH 8.0, 5 mM EDTA, 1 mM DTT, 0.5 M NaCl supplemented with complete protease inhibitor cocktail (Roche Diagnostics, Mannheim, Germany). Upon 15 min of incubation at 4 °C on a rotating platform, extracts were clarified by centrifugation (10 min, 10,000 rpm) and protein content was determined in a final step using Bradford assay. 

Cell fractionation was conducted by cell lysis in buffer containing 10 mM HEPES-KOH pH 7.9, 1.5 mM MgCl_2_ and 10 mM KCl for 15 min on ice. The lysate was then supplemented with 10% NP-40 and vortexed for 30 s. After centrifugation, the cytoplasmic fraction was obtained in the supernatant. Cell pellets were washed using isotonic buffer (10 mM Tris-HCl pH 7.4, 150 mM NaCl) and nuclei were lyzed in buffer containing 25% glycerol, 20 mM HEPES-KOH pH 7.9, 420 mM NaCl, 1.5 mM MgCl_2_, 0.5 mM EDTA. Upon thorough resuspension and incubation for 20 min on ice, nuclear extracts were isolated by centrifugation. Protein content was determined using the Bradford assay.

### 2.4. Co-Immunoprecipitation

Harvested cells were lyzed in buffer (40 mM Tris-HCl pH 8, 1 mM EDTA, 1 mM PMSF) containing 5 mM *N*-ethylmaleimide (NEM) for 10 min on ice. Afterwards, 10 mM cysteine was added in order to inhibit NEM and to avoid further alkylation of amine- and thiol-moieties and cells were sonicated. After centrifugation, equal amounts were saved for input analysis and remaining lysates were pre-clarified by incubation with Protein A/G PLUS agarose (Santa Cruz, Dallas, USA) for 2 h at 4 °C on a rotating platform. Incubation for 2 h at 4 °C with anti-p53 antibody (rabbit polyclonal) followed. Finally, antigen-antibody complexes were captured by incubation with Protein A/G agarose beads overnight at 4 °C. Upon thorough washing, beads were denatured at 95 °C for 5 min using non-reducing Laemmli buffer. Samples were analyzed by SDS-PAGE and western blot analysis as described below.

### 2.5. Transient Transfection with siRNA

Knockdown of ATG5 was performed using siGENOME SMARTpool siRNA purchased from Dharmacon (Lafayette, LA, USA). Non-sense, scrambled siRNA, also purchased from Dharmacon, was used as control. Transfections were carried out as reported previously [33]. Briefly, cells were transfected with 10 nM siRNA using Lipofectamine RNAimax (Invitrogen, Darmstadt, Germany) for 24 h before LA treatment for 48 h. Knockdown of ATG5 was verified by western blot analysis.

### 2.6. SDS-PAGE and Immunoblot Analysis

Western blot analysis was performed as described [34]. Equal protein amounts were separated by SDS-PAGE followed by transfer onto a nitrocellulose membrane (Perkin Elmer, Rodgau, Germany) with a wet blot chamber (BioRad, München, Germany). Afterwards, membranes were blocked with 5% nonfat dry milk in TBS/0.1% Tween-20 for 1 h at RT. Primary antibody incubation was performed overnight at 4 °C followed by 3 × 5 min washing in TBS/0.1% Tween-20. Membranes were incubated with appropriate secondary antibodies for at least 1 h at RT. After 3 × 5 min washing, proteins were detected using Western Lightning® Plus-ECL (Perkin Elmer, Rodgau, Germany).

### 2.7. Preparation of RNA and Quantitative Real Time PCR

Gene expression analysis was essentially performed as described previously [35]. Total RNA was isolated using the NucleoSpin^®^ RNA Kit (Macherey-Nagel, Düren, Germany). RNA concentrations were determined using a NanoDrop^TM^ 2000 spectrophotometer (Thermo Scientific) and 0.5 µg of total RNA was transcribed into cDNA using the Verso cDNA synthesis Kit (Thermo Scientific, Dreieich, Germany). qPCR was performed with the SensiMix^TM^ SYBR Green & Fluorescein Kit (Bioline, London, UK) and the CFX96^TM^ Real-Time PCR Detection System (Biorad, München, Germany) with the primers specified below (Table 1). In all three experiments, RT qPCR was conducted using technical duplicates. The analysis was performed using CFX Manager^TM^ Software. Non-transcribed controls were included in each run. Finally, expression of genes of interest was normalized to *GAPDH* and *ACTB*. The solvent control was set to one. 

### 2.8. Confocal Immunofluorescence Microscopy of p53

Immunofluorescence staining and confocal microscopy was conducted as described previously [36]. Briefly, cells were seeded on cover slips and treated as indicated. Upon fixation with 4% paraformaldehyde, cells were washed with 100 mM glycine. After blocking using 5% bovine serum albumin (BSA) in PBS with 0.3% Triton X-100, samples were incubated with anti-p53 antibody (mouse monoclonal, 1:500 in blocking solution) for 1 h at RT. As secondary antibody goat-anti-mouse coupled with Alexa488 (1:400 in PBS plus 0.3% Triton X-100, 1 h at RT) was used. Nuclei were finally counterstained using TO-PRO-3 (1:100 in PBS). Cover slips were mounted using VectaShield (Vector Labs, Burlingame, USA) and fluorescence microscopy images were taken using a Zeiss AxioObserver Z1 microscope equipped with a confocal LSM710 laser-scanning unit (Zeiss, Oberkochen, Germany). Pictures were analyzed and processed using Image J.

### 2.9. Flow Cytometry-Based Analysis of Autophagy Induction

Using the CytoID^®^ Green Autophagy Detection Kit (Enzo Life Science, Lörrach, Germany), autophagy levels were monitored as reported [33]. According to the manufacturer’s protocol, attached and detached cells were harvested, washed with PBS, and stained for 30 min at 37 °C in the dark in phenol-free medium with 0.1 % dye. After a final washing step, measurement of the samples was carried out using BD Canto II and gating was performed using FACSDiva software (BD Biosciences). Unstained samples were measured with each experiment to subtract autofluorescence.

### 2.10. Cell Death Measurement by FLOW cytometry

Cell death was measured by flow cytometry using AnnexinV/PI staining as previously described [15]. In short, adherent and detached cells were harvested using Trypsin/EDTA, pelleted, washed with PBS, and resuspended in 50 µL binding buffer (10 mM HEPES pH 7.4, 140 mM NaCl, 2.5 mM CaCl2, 0.1% BSA) plus 2,5 µL AnnexinV-FITC (Miltenyi Biotec, Bergisch Gladbach, Germany). Upon 15 min incubation on ice, 430 µL binding buffer and 10 µL propidium iodide (PI; Sigma Aldrich, Munich, Germany; 50 µg/mL) were added and samples were analyzed using a BD Canto II (BD Biosciences, Heidelberg, Germany). Gating of living cells (Annexin V/PI double negative), early apoptotic cells (Annexin V-positive, PI-negative) [37], and late apoptotic/necrotic cells (Annexin V/PI-double positive) [38], as well as data evaluation was performed with BD Diva software.

### 2.11. Determination of Reactive Oxygen Species by Flow Cytometry

Levels of reactive oxygen species (ROS) were quantified using flow cytometry as described [39]. Incubation with 400 µM H_2_O_2_ (Merck, Darmstadt, Germany) for 20 min in PBS served as positive control. Cells were loaded with 2.5 µM CM-H_2_DCFDA (Invitrogen, Darmstadt, Germany) in PBS for 30 min at 37 °C in phenol red- and serum-free medium. Upon washing with PBS, cells were harvested using Trypsin/EDTA, pelleted, resuspended in PBS, and analyzed using a BD Canto II (BD Biosciences, Heidelberg, Germany) and evaluated using BD Diva software.

### 2.12. Assessment of Combination Effect

ATP assays were used to measure cell viability [15]. According to the manufacturer’s protocol of CellTiter-Glo^®^ Luminescent Cell Viability Assay Kit (Promega, Mannheim, Germany), 0.5 × 10³ HCT116 cells per well were seeded in a white 96-well-plate. Drug doses were chosen on the basis of IC_50_ values and a constant ratio recommended for the Chou–Talalay-method [40]. Upon measurement using the luminometer Fluoroskan Ascent FL (Thermo Scientific, Vantaa, Finland), data was processed and evaluated using CompuSyn (ComboSyn Inc., Paramus, NJ, USA) and combination indexes (CI) were calculated.

### 2.13. Statistics

Experiments were performed independently three times, except when otherwise stated. Representative experiments are displayed. Values are presented as means + standard error of the means (SEM) using GraphPad Prism 7.0 software. Statistical analysis was performed using two-sided Student’s *t*-test and statistical significance was defined as *p* < 0.05.

## 3. Results

### 3.1. LA Leads to the Depletion of Wildtype and Mutant p53 in CRC Cell Lines 

**Figure 1:**
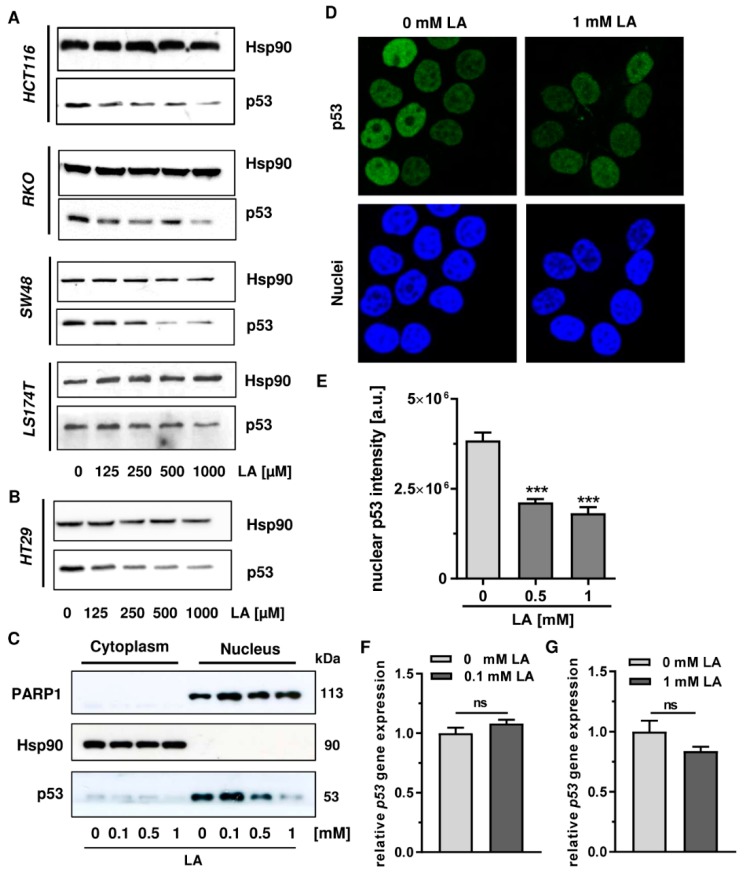
LA triggers depletion of p53 in CRC cells. (**A**) A panel of p53-wild type cells including HCT116, RKO, SW48, and LS174T were treated with increasing doses of LA for 48 h as indicated. EtOH was included as solvent control (0 µM). Depletion of p53 was monitored using western blot analysis. Hsp90 was visualized as loading control. (**B**) The p53-mutated cell line HT29 was exposed to LA and p53 protein expression was analyzed as described in A. (**C**) HCT116 cells treated with increasing doses of LA were collected after 48 h and subjected to cell fractionation. Cytoplasmic and nuclear fractions were separated by SDS-PAGE followed by immunoblot analysis for p53 levels. Hsp90 served as loading control for the cytoplasm, while PARP1 was used as loading control for the nucleus. (**D**) Confocal microscopy of p53 was performed in HCT116 cells upon 48 h of incubation with 0 mM LA (EtOH as solvent control) or 1 mM LA. Fixed cells were stained with a p53-antibody followed by an Alexa488-coupled secondary antibody (displayed in green) and nuclei were counterstained using TO-PRO3 (blue). Representative images are shown. (**E**) Quantification of nuclear p53 intensity as assessed in D. Data is expressed as mean + SEM (*n* = 3, at least 6 sections per group). *** *p* < 0.001. (**F**,**G**) Gene expression of *p53* in LA-treated HCT116 cells using qPCR after 24 h incubation. EtOH was included as solvent control (0 mM). *p53* expression levels are normalized to *ACTB* and *GAPDH*. Data is presented as mean + SEM (*n* = 3). Ns, not significant.

The impact of LA on p53 protein and function has been largely unstudied so far. In our previous work, we provided evidence that cell death induction by LA in CRC cells is independent of p53 and was not accompanied by initial p53 stabilization [15]. In order to investigate the effects of LA on p53 in more detail, we performed western blot analysis of p53 in response to LA treatment in various CRC cell lines. Among a panel of CRC cell lines harbouring wildtype p53 (HCT116, SW48, RKO, LS174T) [41], p53 was depleted in a dose-dependent manner upon incubation with LA for 48 h (Figure 1A). In all cell lines tested, doses as low as 125 µM induced this effect, which was shown to be dose-dependent and reached a maximum at 1 mM LA. While the effect in general was cell line-independent, the overall depletion was most pronounced in HCT116 as well as SW48 cells. The solvent control ethanol (0 µM) did not affect p53 levels in any cell line (Figure 1A). In the same experimental set-up, HT29 cells bearing mutant p53 [41] were incubated with increasing concentrations of LA for 48 h (Figure 1B). As demonstrated for p53 wildtype cells, p53 was depleted in HT29 cells in a comparable and dose-dependent manner. 

In order to analyze LA-triggered depletion of p53 in a subcellular context, cell fractionation was performed following LA treatment. The chaperone Hsp90 localized to the cytoplasm [42] and the nuclear protein PARP-1 involved in the DDR [43] were used to validate proper cell fractionation and equal protein loading. Western blot analysis revealed p53 primarily in the nuclear fraction (Figure 1C), which is in line with the data obtained by confocal immunofluorescence microscopy (Figure 1D). Incubation with 0.5 or 1 mM LA caused a strong, dose-dependent depletion of nuclear p53 protein, which was also observed in cytoplasmic fractions (Figure 1C). This finding was confirmed by confocal microscopy of HCT116 cells exposed to LA for 48 h, revealing a clear decrease of p53 staining in the nucleus as compared to solvent-treated control cells (Figure 1D). Quantitative evaluation of immunofluorescence staining showed a reduction of nuclear p53 protein by approximately 50% at a dose of 1 mM LA (Figure 1E). 

In a next step, we addressed the question whether LA also influences *p53* at the gene expression level. In order to assess mRNA levels of *p53* upon incubation with LA, HCT116 cells were treated with LA for 24 h. Following RNA isolation and cDNA synthesis, *p53* expression was assessed by quantitative real-time PCR using *ACTB* and *GAPDH* as housekeepers. While a dose of 0.1 mM LA slightly increased *p53* expression (Figure 1F), 1 mM LA resulted in a minor, but not significant reduction of *p53* expression (Figure 1G).

**Figure A1:**
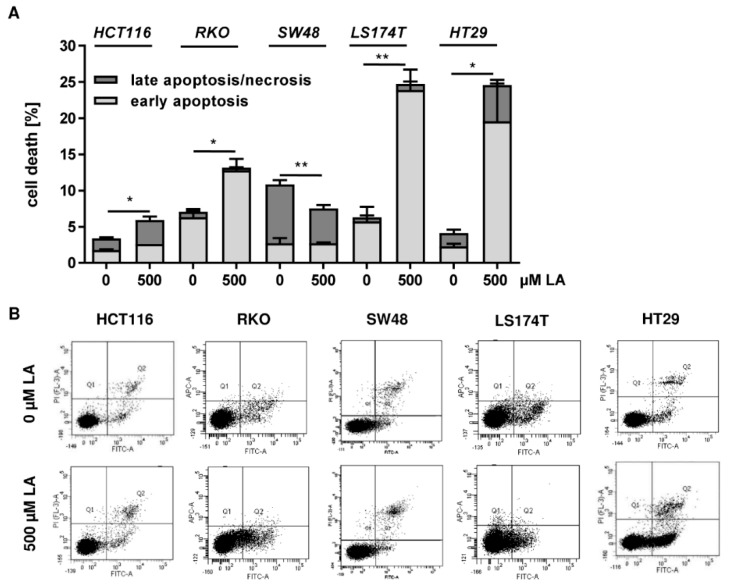
Cytotoxicity of LA in different cell lines after 48 h. (**A**) Flow cytometric cell death measurement using AnnexinV/PI staining in a panel of CRC cells including HCT116, RKO, SW48, LS174T, and HT29. CRC cells were incubated with 500 µM LA or the solvent control EtOH (0 µM) for 48 h. Data is presented as mean + SEM (n ≥ 3). * *p* < 0.05; ** *p* < 0.01. (**B**) Representative dot plots of (A).

In our previous studies, LA was shown to induce cell death and reduced viability in the above-mentioned CRC cell lines with IC_50_ values (72 h) ranging from 266 to 1500 µM [9,15]. To study whether cytotoxic effects already occurred after 48 h, cell death induction was measured by Annexin V/PI staining. In HCT116 and SW48 cells, 500 µM LA had only weak or no cytotoxic effects (Figure A1), but already triggered substantial p53 degradation (Figure 1A). In RKO cells, a moderate cell death induction by LA was noted, while LA induced clear cytotoxicity in LS174T and HT29 cells (Figure A1).

**Figure 2:**
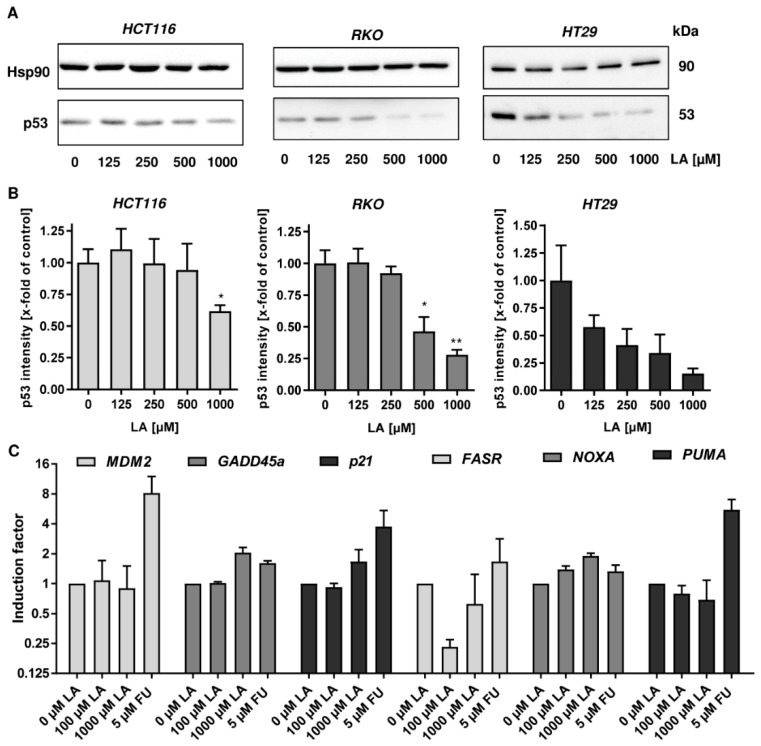
Early p53 depletion in CRC cells and impact on p53-dependent gene expression. (**A**) CRC cells with wildtype p53 (HCT116 and RKO) and mutant p53 (HT29) were treated with increasing doses of LA for 24 h as indicated. EtOH was included as solvent control (0 µM). Depletion of p53 was monitored using western blot analysis. Hsp90 was visualized as loading control. (**B**) Densitometric analysis of data presented in A. p53 levels were normalized to Hsp90. Data is presented as mean + SEM (*n* = 3). * *p* < 0.05; ** *p* < 0.01. (**C**) Gene expression of p53 targets genes (*MDM2*, *GADD45a*, *p21*, *FASR*, *NOXA*, and *PUMA*) in HCT116 cells treated with LA for 24 h determined by qPCR analysis. EtOH served as solvent control (0 µM LA), while 5-FU was used as positive control. Gene expression levels are normalized to *ACTB* and *GAPDH*. Data is presented as mean + SEM (*n* = 3), except for *FASR* analysis (*n* = 2).

To further address this issue, p53 levels were assessed by western blot analysis already after 24 h of LA exposure. In all three CRC cell lines (HCT116, RKO, and HT29) LA provoked p53 depletion in a dose-dependent manner (Figure 2A,B). Furthermore, expression of p53-related genes was determined by quantitative real time PCR in HCT1116 cells. The positive control 5-FU, an antimetabolite used in CRC therapy, triggered a transcriptional p53 response as reflected by strong induction of the ubiquitin ligase *MDM2*, the cell cycle regulator *p21* and the apoptosis factor *PUMA*, while other apoptosis-related genes, *FASR* and *NOXA*, were only slightly induced (Figure 2C). In turn, both low and high doses of LA did not affect *MDM2* expression and slightly downregulated the expression of the pro-apoptotic genes *PUMA and FASR*. Only at a high dose (1000 µM) LA moderately stimulated *GADD45* and *p21* expression (Figure 2C), which are both implicated in cell cycle regulation. 

In conclusion, both wildtype and mutant p53 were universally depleted on the protein level among various CRC cell lines after LA treatment in a time- and dose-dependent manner. This occurred already to a substantial extent after 24 h and thus preceded LA-triggered cytotoxicity. Moreover, LA hardly influenced neither *p53* gene expression nor the expression of well-known p53 target genes, while 5-FU clearly induced a p53 transcriptional response. 

### 3.2. Supplementation with Antioxidants Does Not Rescue p53 Depletion

**Figure 3:**
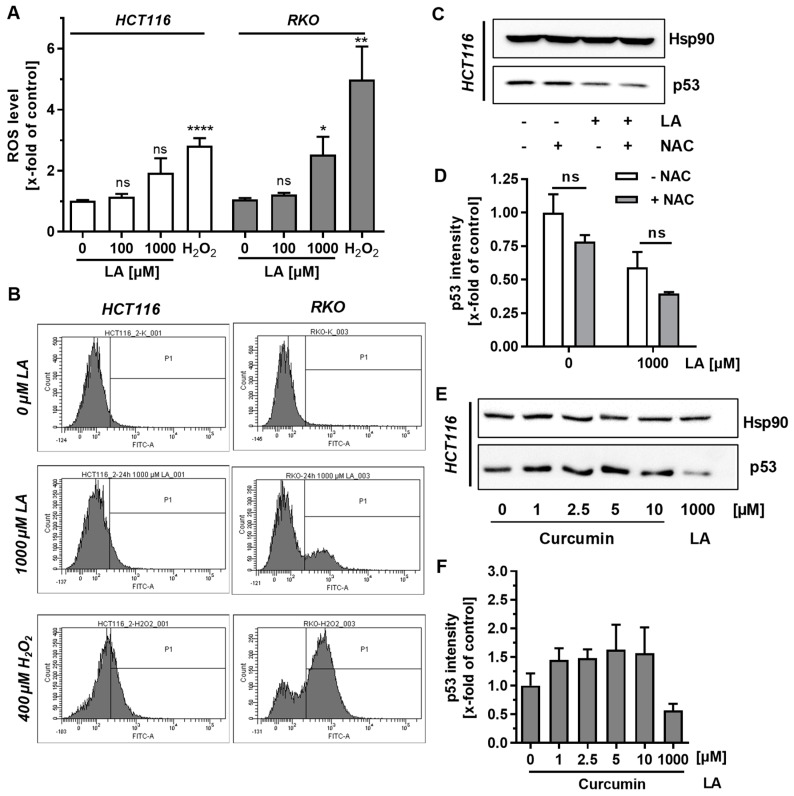
Induction of ROS upon LA treatment and impact of antioxidants on p53 depletion. (**A**) Flow cytometric determination of reactive oxygen species (ROS) in HCT116 and RKO cells upon LA treatment for 24 h using the CM-H_2_DCFDA dye. EtOH was used as solvent control (0 µM LA). H_2_O_2_ (400 µM, 20 min) served as positive control. Data is presented as mean + SEM (*n* = 5). Ns: *p* > 0.05; * *p* < 0.05; ** *p* < 0.01; **** *p* < 0.0001. (**B**) Representative histograms of A. (**C**) HCT116 cells were treated with 1 mM LA for 24 h either with or without co-incubation with the antioxidant NAC (5 mM). Cells were lyzed and subjected to SDS-PAGE, followed by western blot analysis of p53. Hsp90 was visualized as loading control. (**D**) Densitometric analysis of p53 signals obtained in C following normalization to Hsp90. Data are presented as mean + SEM (*n* = 3). Ns: *p* > 0.05. (**E**) HCT116 cells were treated with increasing doses of curcumin (1–10 µM) for 48 h and p53 levels were analyzed using western blot analysis. Hsp90 served as loading control. (**F**) Densitometric analysis of data presented in E. p53 signals were normalized to Hsp90. Data are presented as mean + SEM (*n* = 3).

LA was shown to exert pro-oxidative effects in some tumor cell lines [16,17,31] and may also directly modify reactive protein thiols [13]. In order to address the contribution of these processes to LA-mediated depletion of p53, we first measured induction of ROS by flow cytometry. H_2_O_2_ was included as positive control, which clearly increased ROS levels (Figure 3A,B). While 100 µM LA had no effect on cellular ROS formation, 1000 µM LA approximately doubled the ROS burden in both HCT116 and RKO cells, however only the latter was statistically significant (Figure 3A,B). 

Next, the effect of the thiol-containing antioxidant NAC on p53 level was studied in HCT116 cells following LA treatment. Interestingly, NAC single treatment slightly, but not significantly diminished p53 levels (Figure 3C,D). As already shown in Figure 2, LA caused p53 depletion, which was however not reverted by co-incubation with NAC (Figure 3C,D). Furthermore, the impact of curcumin on p53 was assessed, which is a natural phenolic compound and antioxidant [44]. HCT116 cells were treated with up to 10 µM curcumin for 48 h, which resulted in a moderate p53 accumulation already at 1 µM (Figure 3E,F). This induction remained constant at higher curcumin concentrations, although ≥ 5 µM curcumin displayed enhanced cytotoxicity with strongly reduced cell density and increased cell rounding (data not shown).

In summary, LA caused a moderate ROS induction at high doses, which coincided with p53 depletion. However, the thiol-containing antioxidant NAC was not able to revert this effect. In contrast, the phenolic antioxidant curcumin even enhanced p53 levels, pointing towards an intrinsic, structure-dependent effect of LA on p53. 

### 3.3. LA-Triggered Autophagy Is Not Involved in the Mechanism of p53 Depletion

**Figure 4:**
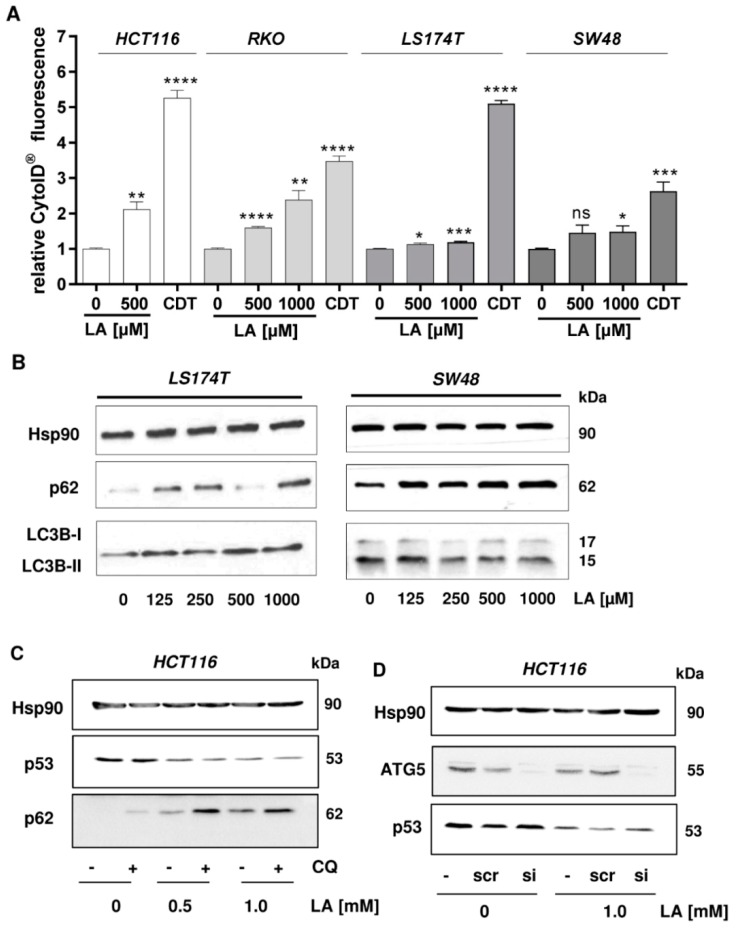
Autophagosome formation and p62 accumulation by LA in CRC cells and contribution of autophagy to p53 depletion. (**A**) Relative autophagosome levels in HCT116, RKO, LS174T, and SW48 cells after 48 h of LA treatment determined by the CytoID® Autophagy Detection Kit and flow cytometry. EtOH was used as solvent control (0 µM LA). Cytolethal distending toxin (CDT) served as positive control. Data are shown as mean + SEM (*n* = 4). ns *p* > 0.05; * *p* < 0.05; ** *p* < 0.01; *** *p* < 0.001; **** *p* < 0.0001. (**B**) The autophagy marker LC3B and accumulation of the autophagy receptor p62 were visualized by western blot analysis of LS174T and SW48 cells treated with increasing doses of LA for 48 h. Hsp90 served as loading control. (**C**) Pharmacological inhibition of autophagy and impact of LA on p53 status. HCT116 cells were incubated with LA for 48 h as indicated. CQ was added 16 h prior to harvesting of the cells. Whole-cell extracts were separated by SDS-PAGE followed by western blot analysis of p53 and p62. Hsp90 served as loading control. (**D**) Genetic abrogation of autophagy and p53 depletion by LA. HCT116 cells were transiently transfected with ATG5 siRNA or scrambled (scr) siRNA. Cells were then incubated with 1 mM LA or the solvent control EtOH (0 mM LA). Samples were subjected to SDS-PAGE and western blot analysis of p53. Successful knockdown was confirmed by immunoblot detection of ATG5, while Hsp90 was used as loading control.

**Figure A2:**
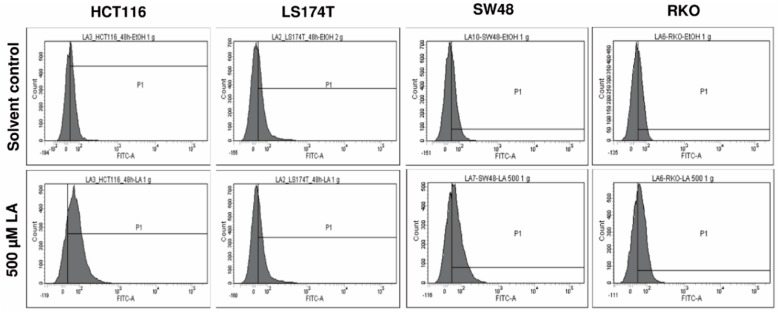
Effect of LA on autophagosome formation in different CRC cells. Cells were treated with LA or EtOH as solvent control for 48 h, harvested and stained using the CytoID® Green Detection kit. Cells were then analyzed by flow cytometry. Representative histograms are shown.

Since our previous study demonstrated that LA stimulates autophagy [13] as a cell survival pathway, we hypothesized that this process may be involved in the depletion of p53. The highly regulated process of (macro-)autophagy describes the targeted digestion and recycling of proteins and organelles that are unneeded, dysfunctional, or required as nutrient source under cellular stress conditions—e.g., starvation or oxidative stress [45]. During autophagy targeted substrates are engulfed in double-membraned vesicles called autophagosomes, which fuse with hydrolases-filled lysosomes to form autolysosomes [46,47]. In order to test our hypothesis, we first studied the formation of autophagic vesicles in CRC cells exposed to LA using the CytoID® Detection Kit. The bacterial genotoxin cytolethal distending toxin (CDT) was used as positive control, since it was recently described as autophagy inducer in CRC cells [33]. Flow cytometry-based measurements showed the strongest induction of autophagosome formation in HCT116 cells upon treatment with 500 µM LA (Figure 4A, first panel). Similar levels could be reached in RKO cells using a dose of 1000 µM LA, while 500 µM LA showed weaker effects on autophagy (Figure 4A, second panel). A very low, but statistically significant increase of autophagic vesicles was measurable in LS174T cells at the highest LA dose. The same trend of induction, without reaching statistical significance, was observed in SW48 cells. Representative histograms are displayed in Figure A2. To detail these findings, the conversion of LC3B-I to LC3B-II, which is part of the autophagosome, was monitored by western blot analysis. While LA caused a modest increase in LC3B-II formation in LS174T cells, a reduction of LC3B-II was detected in SW48 cells (Figure 4B), suggesting that autophagy is only a minor pathway triggered by LA in these two CRC cell lines. In turn, LA was shown to significantly increase LC3B-II formation in HCT116 and RKO cells by promoting the autophagic flux as demonstrated by chemical autophagy inhibitors in our previous study [13]. The autophagy receptor p62, which is also a substrate of autophagy, was analyzed as well, showing a dose-dependent p62 accumulation after LA treatment in both SW48 and LS174T cells (Figure 4B). This goes in line with our previously published work, which also revealed a strong LA-dependent p62 induction in HCT116 and RKO cells [13]. Taken together, LA triggers autophagosome formation in a rather cell line-specific manner, whereas p62 accumulation occurred in all tested cell lines. To further elucidate the impact of LA on the autophagic flux in LS174T and SW48 cells, chemical autophagy inhibitors could be used.

To further study the possible interplay of autophagy induction and p53 depletion, we focused on HCT116 cells and monitored p53 levels with simultaneous inhibition of autophagy. To this end, autophagy was abrogated either by the pharmacological inhibitor chloroquine (CQ) or via siRNA knockdown of the key regulator protein ATG5. Upon activation by ATG7, ATG5 builds a complex with ATG12 and ATG16 to elongate the phagophore in autophagic vesicles and catalyzes the lipidation and the conversion of LC3B-I to LC3B-II, representing an early event in autophagy [48]. When cells were co-incubated with CQ, p62 levels further increased in all samples indicating successful abrogation of autophagy. Nevertheless, depletion of p53 was unaffected when HCT116 cells were co-treated with 0.5 or 1 mM LA and CQ (Figure 4C), arguing against a contribution of the autophagosomal–lysosomal degradation pathway. Furthermore, siRNA-mediated knockdown of ATG5 was performed in HCT116 cells. Western blot analysis confirmed the successful knockdown with minimal residual levels of ATG5. However, LA-triggered p53 depletion was not rescued by ATG5 knockdown, further excluding a contribution of autophagy to the observed p53 degradation (Figure 4D). Collectively, the results provide evidence that the autophagic machinery is not involved in the depletion of p53 following LA treatment.

### 3.4. LA Causes Nrf2 Induction, Which is Dispensible for p53 Degradation

**Figure 5:**
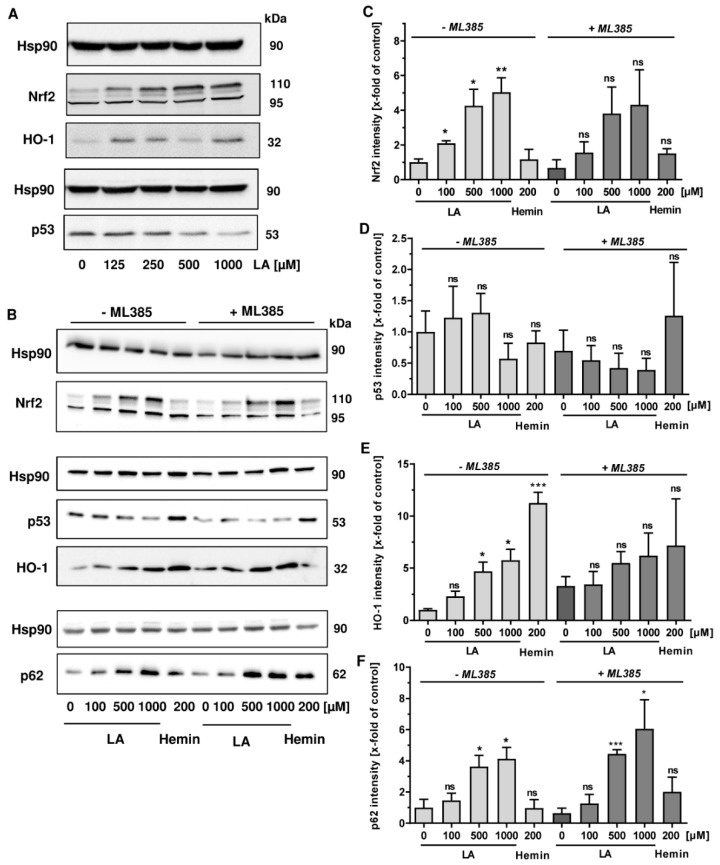
LA induces Nrf2, which is likely dispensable for p53 degradation. (**A**) HCT116 cells were incubated with increasing doses of LA (125–1000 µM) for 48 h. Cells were thereafter subjected to SDS-PAGE and western blot analysis of p53, Nrf2 and its downstream target HO-1. EtOH (0 µM) served as solvent control. Hsp90 was visualized as loading control. (**B**) HCT116 cells were incubated for 48 h with LA in the presence or the absence of ML385, a pharmacological Nrf2 inhibitor. Hemin (200 µM, 24 h) was included as positive control for HO-1 induction. EtOH (0 µM) served as vehicle control. Cells were then lyzed and underwent western blot analysis of Nrf2, p53, HO-1, as well as p62. Hsp90 was used as loading control. (**C**–**F**) Densitometric quantification of NRF2 (C), p53 (D), HO-1 (E) and p62 (F) obtained from three independent experiments as described and shown in B. Data are given as mean + SEM (*n* = 3). ns *p* > 0.05; * *p* < 0.05; ** *p* < 0.01; *** *p* < 0.001.

As a next step, the role of nuclear factor E2-related factor (Nrf2) in LA-triggered p53 degradation was analyzed. Nrf2 is an important transcription factor, which is activated upon oxidative stress and by electrophilic compounds, driving the expression of genes involved in the antioxidative response and detoxification [49]. Interestingly, it has also been linked to the expression of the autophagy receptor p62, which was shown to be upregulated by LA herein (Figure 4B,C). Western blot analysis revealed that LA causes a dose-dependent accumulation of Nrf2 (Figure 5A, top panel), which migrates at 95 and 110 kDa as reported [50]. At the same time, its downstream target heme oxygenase 1 (HO-1) was upregulated (Figure 5A, middle panel), while p53 was depleted as observed before (Figure 5A, bottom panel). To determine the contribution of Nrf2, we made use of a specific small molecule inhibitor of Nrf2 called ML385, which disrupts the binding of Nrf2 to its antioxidant response element (ARE) in the promoter region of Nrf2-reguated genes [51]. Consistent with the data obtained before, LA treatment resulted in a dose-dependent induction of Nrf2 (Figure 5B,C, left panel). The Nrf2 inhibitor ML385 slightly reduced Nrf2 levels in all samples irrespective of the treatment (Figure 5B,C, right panel). The Nrf2 inhibitor did not rescue the LA-dependent p53 depletion, but slightly promoted p53 degradation, which was already visible in the absence of LA (Figure 5B,D). The Nrf2 target HO-1 was moderately induced by LA, while it was almost unaffected by the inhibitor. Hemin treatment provoked a robust induction of HO-1 as expected [52], which was significantly blocked under Nrf2 inhibition (Figure 5B,E). Finally, p62 levels were assessed and revealed a further, but rather small increase, in p62 upon Nrf2 inhibition (Figure 5B,F). Altogether, Nrf2 is upregulated by LA in a dose-dependent manner, which coincided with p53 depletion. However, Nrf2 stabilization was not linked to p53 degradation as illustrated by the Nrf2 inhibitor ML385.

### 3.5. p53 is Readily Ubiquitinated upon LA Treatment

**Figure 6:**
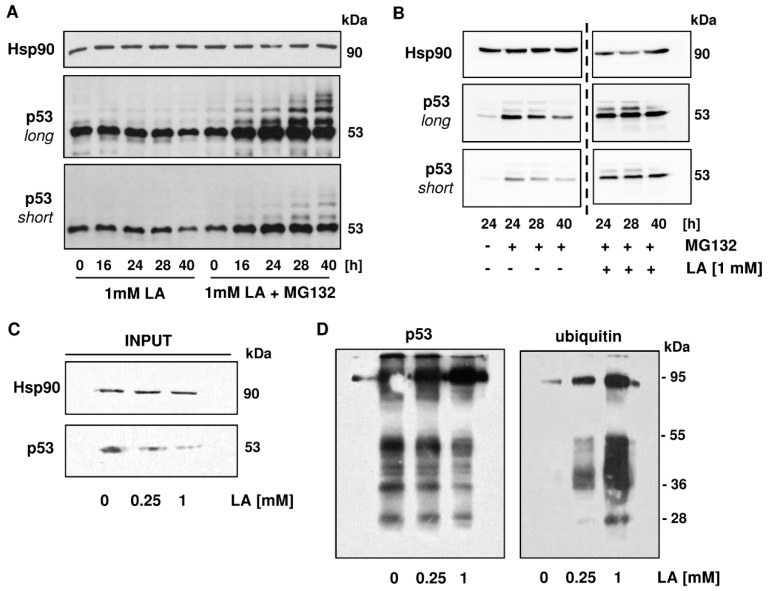
LA causes enhanced ubiquitination of p53. (**A**) Enhanced ubiquitination as shown in HCT116 cells over a time period of 16 to 40 h using co-treatment with the proteasome inhibitor MG132 (10 µM), which was supplemented 16 h after treatment with 1 mM of LA. Cells were harvested at indicated time points and underwent western blot analysis. (**B**) Higher levels of p53 ubiquitination in HCT116 cells upon co-treatment of LA with MG132 after 24, 28, and 40 h with the corresponding MG132-control. Experiment was conducted as described in A. Samples were analyzed on the same blot membrane and cut for better visualization (dashed line). (**C**,**D**) Co-immunoprecipitation of p53 in HCT116 after 48 h of LA-treatment. EtOH served as solvent control (0 mM LA). The left panel describes input for co-immunoprecipitation with rabbit polyclonal p53-antibody. The right panel represents output for mouse monoclonal p53-antibody and mouse monoclonal ubiquitin-antibody.

Another major pathway for cellular protein homeostasis is the ubiquitin-proteasome pathway, in which substrate proteins are covalently labeled with ubiquitin moieties and channelled into the proteasome for degradation, thereby giving rise to recyclable amino acids [53]. Basal p53 levels are tightly controlled by ubiquitination, which is catalyzed by the E3 ubiquitin ligase MDM2 together with Mdmx [54]. First, ubiquitination of p53 was analyzed after treatment with LA in the absence or presence of the proteasome inhibitor MG132. As shown above, 1 mM LA caused a time-dependent depletion of p53 in HCT116 cells. Interestingly, addition of the proteasome inhibitor after 16 h of LA treatment resulted in the accumulation of p53 and revealed the presence of mono- and polyubiquitinated p53 species, which migrated at a higher molecular weight as unmodified p53, forming the typical ubiquitin laddering (Figure 6A). As pointed out above, p53 has a high basal proteasomal turnover. Therefore, we wished to assess the effects of MG132 with or without LA treatment in order to distinguish between basal and LA-dependent ubiquitination of p53. As expected, single treatment of HCT116 cells with MG132 increased p53 levels and caused the accumulation of ubiquitin-labeled p53 species (Figure 6B, left panel). Intriguingly, the co-treatment of 1 mM LA and MG132 led to a further increase of p53 and its ubiquitinated forms, which was particularly obvious after 40 h (Figure 6B, right panel). It should be noted that a prolonged incubation with MG132 seems to decrease p53 levels in cells after 40 h irrespective of LA treatment or not (Figure 6A,B), which may be attributable to toxic side effects of the inhibitor.

In the next step, cells were treated with LA and p53 immunoprecipitation was performed using a rabbit polyclonal antibody to specifically detect ubiquitin-modified p53. Equal amounts of protein, as determined using the Bradford assay, were subjected to the immunoprecipitation. Western blot analysis of p53 in the prepared whole-cell lysates for immunoprecipitation (input) confirmed depletion of p53 by LA and further showed equal Hsp90 levels in all samples used (Figure 6C). Precipitated p53 was then analyzed by SDS-PAGE under non-reducing conditions and western blot detection with a mouse monoclonal antibody against ubiquitin, revealing a LA-dependent increase in ubiquitinated species, predominantly at about 95 kDa and between 36 and 55 kDa (Figure 6D, right panel). Interestingly, detection of p53 with a mouse monoclonal antibody showed a decrease of p53 protein and some lower molecular weight species, probably reflecting p53 degradation products (Figure 6D, left panel). Taken together, our results show enhanced ubiquitination of p53 following LA treatment and mechanistically linked the proteasomal degradation machinery to the observed p53 depletion in HCT116 cells. 

### 3.6. MDM2 Inhibition Does Not Rescue LA-Triggered p53 Degradation

**Figure 7:**
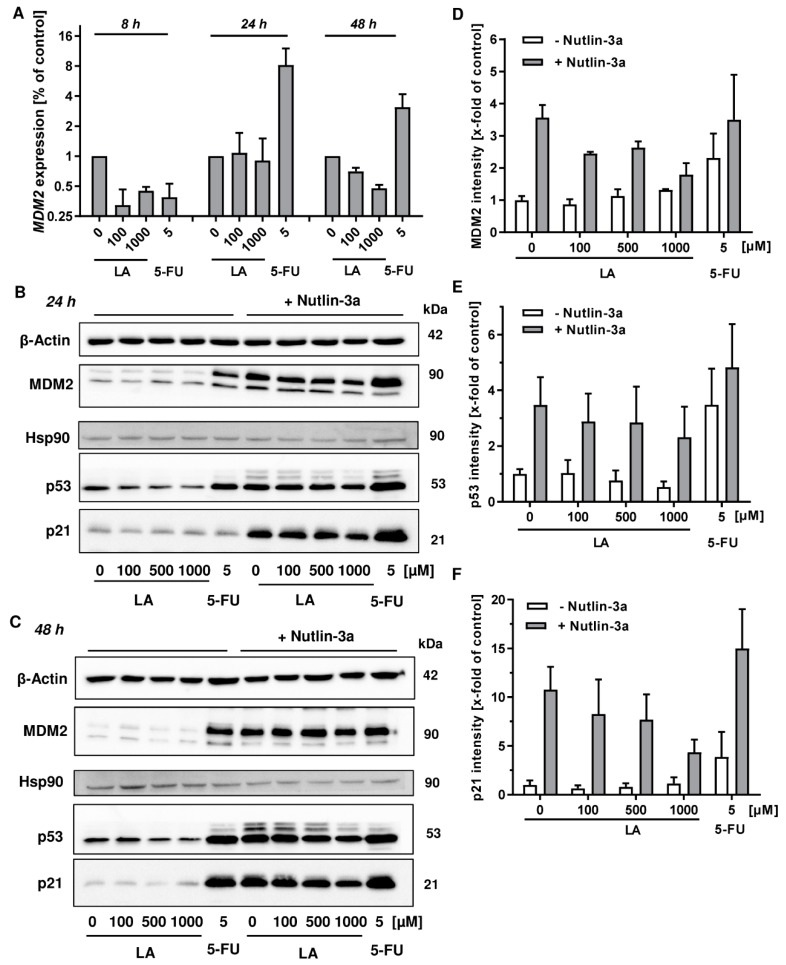
Role of MDM2 in LA-triggered p53-depletion in HCT116 cells. (**A**) Time course of *MDM2* gene expression. Cells were exposed to LA as indicated. EtOH was used as solvent control (0 µM LA), while 5-FU was used as positive control. (**B**,**C**) HCT116 were treated with increasing doses of LA for 24 h (B) or 48 h (C) either with or without the MDM2 inhibitor Nutlin-3a. In addition, 5-FU (5 µM) was used as positive control for p53 and p53-dependent MDM2 and p21 induction. Cells were analyzed by SDS-PAGE followed by western blot detection of MDM2, p53 and p21. Either Hsp90 or β-Actin served as loading control. (**D**–**F**) Densitometric quantification of MDM2 (D), p53 (E), and p21 (F) normalized to the respective loading controls obtained from three independent experiments as described and shown in (B). Data are displayed as mean + SEM of three independent experiments.

In order to dissect the role of MDM2 in LA-triggered p53 ubiquitination and subsequent proteosomal degradation, the time-dependent expression of *MDM2* was monitored by qPCR following LA treatment in HCT116 cells. 8 h after LA treatment, both LA and the anticancer drug 5-FU, which was used as positive control for the p53 transcriptional response, caused a downregulation of *MDM2* (Figure 7A). Interestingly, this negative effect on *MDM2* disappeared in case of LA after 24 h, whereas 5-FU induced a strong upregulation of *MDM2*, which was maintained up to 48 h (Figure 7A). In contrast, LA slightly suppressed *MDM2* expression after 48 h, similar to the short incubation period of 8 h. Next, we made use of the pharmacological MDM2 inhibitor Nutlin-3a. This compound binds to the N-terminus of MDM2 and thereby abrogates its interaction with the N-terminal transactivation domain of p53 [55]. In the absence of Nutlin-3a, incubation of HCT116 cells with LA for 24 h provoked a clear dose-dependent depletion of p53, without affecting MDM2 and p21 protein levels (Figure 7B, left panel, and Figure 7D–F). As expected, 5-FU caused a stabilization of p53 and concomitant induction of its negative regulator MDM2, which matches the gene expression measurements (Figure 7A,B, left panel). In the presence of the MDM2 inhibitor Nutlin-3a, a strong upregulation of the p53 transcriptional response occurred in all samples, irrespective of the treatment (Figure 7B, right panel). Interestingly, p53 levels decreased in a LA-dependent manner as observed before in the absence of Nutlin-3a. In accordance with this finding, the transcriptional targets MDM2 and p21 were also dose-dependently reduced by LA (Figure 7B, right panel and 7D–F). Exposure to LA for 48 h without Nutlin-3a resulted in gradual depletion of p53, while MDM2 or p21 were hardly affected (Figure 7C, left panel). Co-incubation with Nutlin-3a boosted p53 accumulation and its transcriptional response in all treatment groups (solvent control, LA and 5-FU). Nevertheless, LA triggered a dose-dependent decrease of p53 and its downstream target p21 as detected after 24 h (Figure 7C, right panel). It should also be noted that in the presence of Nutlin-3a, ubiquitinated p53 species were clearly visible, forming the typical ubiquitin ladder (Figure 7B,C, right panel). In summary, LA does not upregulate the expression of the E3 ubiquin ligase and negative p53 regulator MDM2. Even in the presence of Nutlin-3a, LA decreased the level of p53 and its downstream target, p21. 

### 3.7. LA Synergizes with Standard Chemotherapeutics by Potentiating Cytotoxicity

Having demonstrated the depletion of p53 in various CRC cell lines bearing wildtype or mutant p53, we aimed to characterize the feasible benefit of combining standard antineoplastic drugs with LA. Standard antineoplastic drugs in cancer therapy of solid tumors include the antimetabolite 5-FU as a mainstay in the therapy of CRC and the anthracycline doxorubicin (Doxo) used for the treatment of a variety of cancers including Hodgkin’s lymphoma and breast cancer [56]. Generally, DNA damaging antineoplastic drugs including 5-FU and Doxo cause stabilization and accumulation of p53, which controls downstream pathways such as DNA repair, cell cycle arrest, apoptotic cell death, and also cell survival mechanisms [21,57]. We speculated that a pre-treatment with LA would deplete p53, attenuate downstream effects such as induction of the cell cycle regulator p21, and thereby drive cancer cells with a high genotoxic load into cell death. 

**Figure 8:**
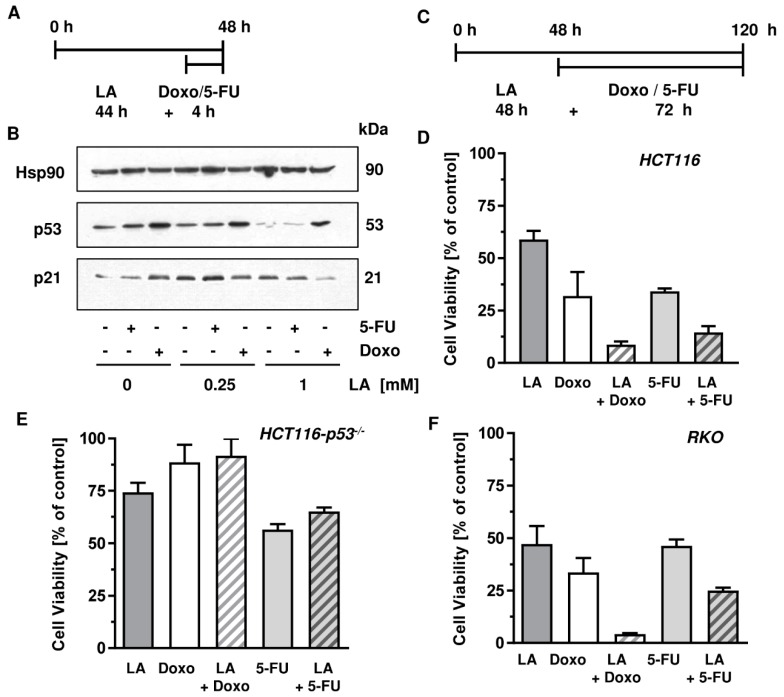
LA attenuates p53 stabilization upon genotoxic stress and synergizes with Doxo and 5-FU. (**A**,**B**) Combination of LA with Doxo or 5-FU, respectively, in HCT116 cells and effects on p53 and its downstream target p21. Cells were treated as indicated in A and harvested after 48 h. Samples were then analyzed with respect to p53 and p21 by SDS-PAGE and western blot. Hsp90 served as loading control. (**C**) Treatment scheme of ATP assays in HCT116, HCT116-p53^−/−^, and RKO cells to determine the combination index. Cells were pre-treated with LA for 48 h and then supplemented with either Doxo or 5-FU. (**D**–**F**) Representative combination effects of LA and Doxo or 5-FU as measured using ATP assays according to the treatment scheme depicted in (**C**) in HCT116 ((**D**); 1 mM LA, 10 µM 5-FU, 2 µM Doxo), HCT116-p53^−/−^ ((**E**); 500 µM LA, 80 µM 5-FU; 62.5 nM Doxo), and RKO ((**F**); 400 µM LA, 5 µM 5-FU, 0.25 µM Doxo) cells.

First, HCT116 cells were treated for 44 h with 1 mM LA followed by a treatment with 5-FU or Doxo for 4 h (Figure 8A). After the total incubation of 48 h, cells were harvested and subjected to western blot analysis of p53. As expected, both 5-FU and Doxo single treatments induced stabilization of p53, which was more pronounced after Doxo incubation (Figure 8B). However, pre-treatment of HCT116 cells with 1 mM LA markedly impaired p53 stabilization following treatment with the anticancer drugs. This effect was very prominent in combination with 5-FU, showing almost no 5-FU-dependent p53 accumulation (Figure 8B). Interestingly, this effect was also visible when applying a dose of 250 µM LA, albeit to a lesser extent. The cell cycle regulator p21 as a downstream target of p53 was especially decreased in cells treated with the combination of Doxo and 1 mM LA, but was only slightly affected in cells co-treated with 5-FU and 1 mM LA (Figure 8B).

**Figure A3:**
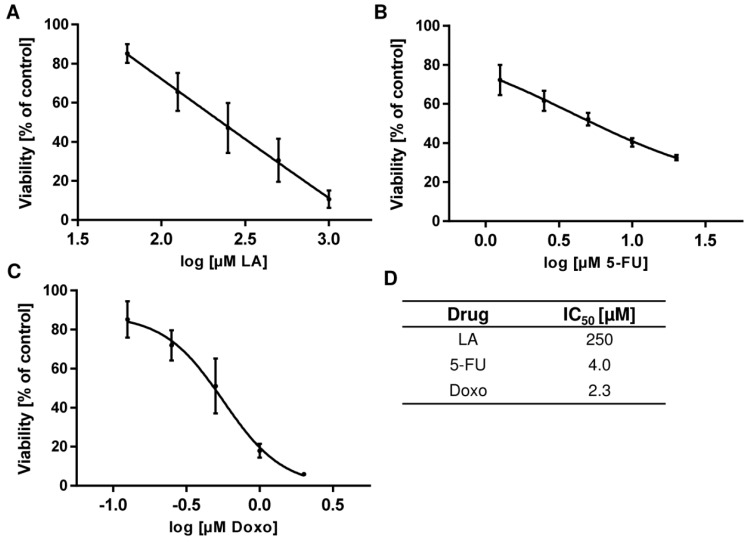
Determination of drug concentrations for application of the Chou Talalay method. HCT116 cells were treated with increasing fixed-ratio doses of LA (**A**) for 120 h; 5-FU (**B**), or Doxo (**C**) for 72 h respectively. Cell viability was measured using ATP assays and normalized to solvent treated control cells. Drug concentrations were transformed into log-scale and plotted against the cell viability. The curve was fitted by non-linear regression with variable slope, providing the IC_50_-values. Data are presented as mean + SEM, *n* = 2, triplicates. (**D**) Summary of IC_50_-values.

**Figure A4:**
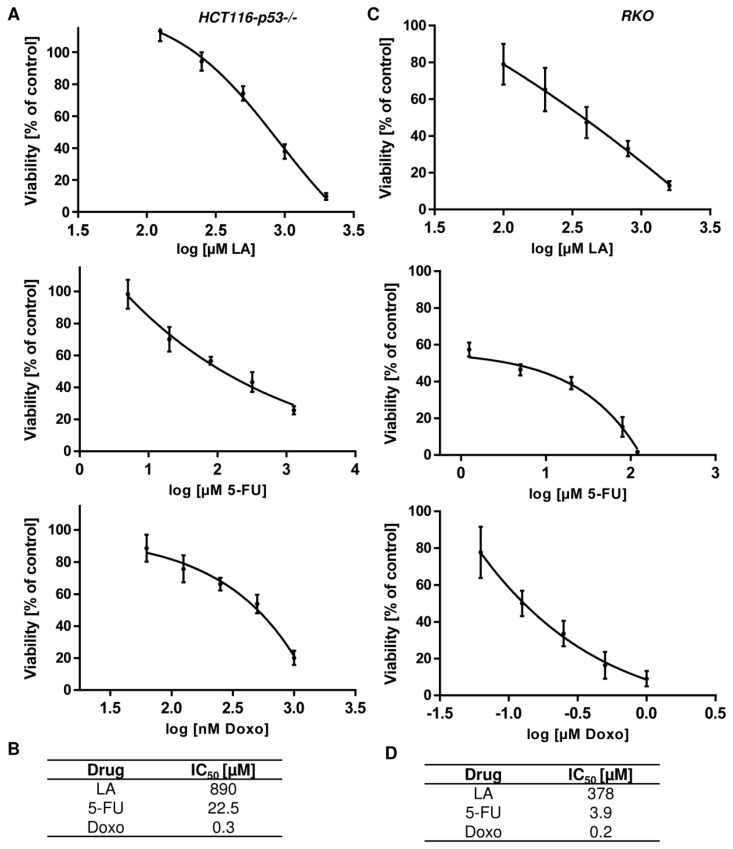
Determination of drug concentrations for application of the Chou–Talalay method. HCT116-p53^−/−^ (left panel) and RKO (right panel) cells were treated with increasing fixed-ratio doses of (**A**) LA for 120 h or 5-FU/Doxo for 72 h respectively. Cell viability was measured using ATP assays and normalized to solvent treated control cells. Drug concentrations were transformed into log-scale and plotted against the cell viability. The curve was fitted by non-linear regression with variable slope, providing the IC_50_-values. Data are presented as mean + SEM, *n* = 2, triplicates. (**B**) Summary of IC_50_-values.

Second, the Chou–Talalay-method was applied in ATP viability assays in order to determine the putative synergism upon combination treatment. Prior to these experiments, IC_50_ values for single treatment with LA, 5-FU, and Doxo had to be determined in order to be able to apply combination regimens in constant ratios based on these calculated IC_50_ values (Figure A3 and Figure A4). The treatment scheme of the conducted ATP viability assays used for the Chou–Talalay-method is depicted in Figure 8C. Cells were incubated with LA in constant ratios for 48 h followed by a 72 h treatment with 5-FU or Doxo in constant ratios (total treatment time: 120 h). Combination treatment of HCT116 cells with LA and Doxo led to a dramatic decrease in viability below 10%, whereas single treatment only displayed moderate-to-high cytotoxicity of 60 and 35%, respectively. Consistent with these findings, the combination of 5-FU and LA peaked in cell viability below 15%, which was clearly more effective than the respective mono-treatments (Figure 8D). Subsequently, the combination indices (CI) were calculated using the Chou–Talalay-method [40] to be 0.55 (LA + Doxo) and 0.67 (LA + 5-FU) respectively (Table 2), which is below 1 for both combination regimens and, thus, designate synergism. Furthermore, the same treatment regimen was applied to p53-deficient HCT116 cells to assess the relevance of LA-mediated p53 depletion for the observed synergistic effects. However, neither the combination of 5-FU and LA nor the combination of Doxo and LA resulted in synergistic cell killing (Figure 8E), but slightly increased viability as compared to the single treatments with the anticancer drugs. This rather antagonistic effect on cytotoxicity was substantiated by the calculated CI using the Chou–Talalay-method, which were for both combinations above 1 and thus indicate antagonism (Table 2). Finally, the combination regimes were tested in RKO cells, which express wildtype p53. Strikingly, both 5-FU and Doxo synergized with LA, which was evidenced by a strong reduction in cell viability compared to the single treatment with 5-FU or Doxo (Figure 8F). In line with these findings, Chou–Talalay analyses demonstrated synergism with CI values of 0.84 (LA + 5-FU) and 0.59 (LA + Doxo), respectively (Table 2). 

**Figure 9:**
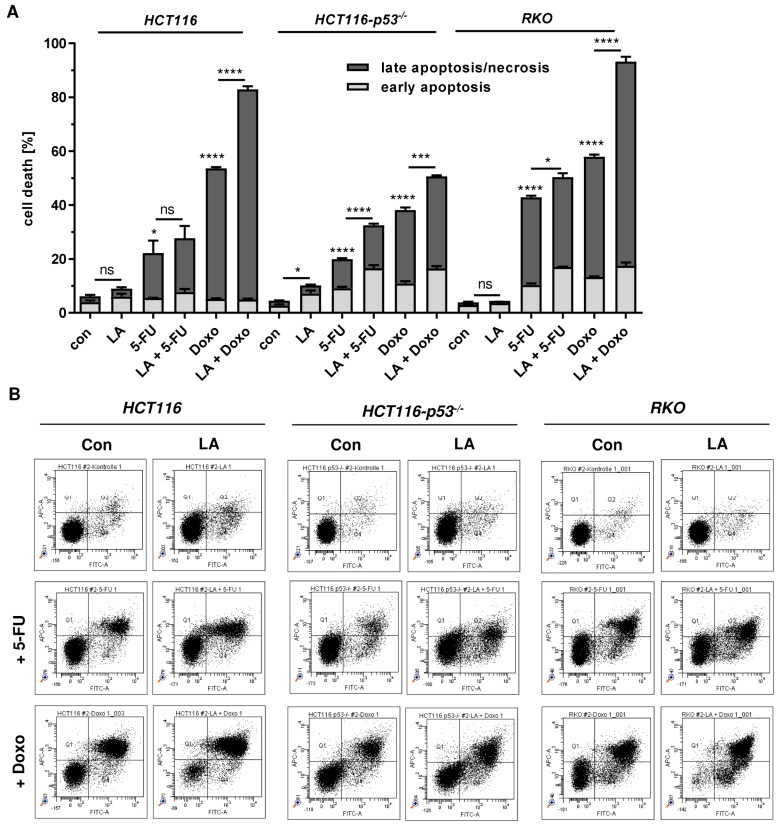
LA potentiates cell death induction in CRC upon treatment with the anticancer drugs Doxo and 5-FU. (**A**) Cells were pre-treated with LA for 48 h (250 µM for HCT116 and HCT116-p53^−/−^; 100 µM for RKO), then supplemented with either Doxo (1 µM for HCT116 and RKO; 5 µM for HCT116-p53^−/−^) or 5-FU (5 µM for HCT116; 20 µM for HCT116-p53^−/−^; 10 µM for RKO) and treated for another 72 h. After 120 h, cells were collected for cell death measurements and subjected to AnnexinV /PI staining followed by flow cytometry. Data is presented as mean + SEM (n≥3). Ns: *p* > 0.05; * *p* < 0.05; *** *p* < 0.001; **** *p* < 0.0001. (**B**) Representative dot plots for experiments shown in (A).

Furthermore, cell death analysis was conducted under the conditions used above. In HCT116 cells, LA at the used dose (250 µM) moderately increased 5-FU-dependent cell death, but strongly increased Doxo-triggered cell death (Figure 9A,B, left panel). In p53-deficient HCT116 cells, LA at the used dose (250 µM) moderately increased cell death induction in combination with both 5-FU and Doxo (Figure 9A,B, middle panel). In RKO cells, LA at the used dose (100 µM) moderately enhanced 5-FU-dependent cytotoxicity, but strongly increased cell death in combination with Doxo, which is similar to the findings obtained in HCT116 cells (Figure 9A,B, left panel vs. right panel). Taken together, LA attenuates the p53 response following exposure to anticancer drugs and induces synergistic cytotoxicity with anticancer drugs, particularly Doxo, in CRC cells in a p53-dependent manner.

## 4. Discussion

The present work shows that the disulphide compound LA triggers ubiquitin-proteasome mediated degradation of p53 in various CRC cell lines and synergizes with DNA damaging antineoplastic drugs (doxorubicin, 5-FU), which usually cause stabilization of p53. First, we provide evidence that p53 is efficiently depleted by LA in a dose-dependent manner. Remarkably, this effect was not limited to wildtype p53, but also occurred in HT29 cells (Figure 1) that express mutated p53 (R273H) [58]. This is a hot spot mutation found within the central DNA binding domain (DBD) and confers a gain-of-function to the mutated p53 protein [59]. The R273H mutant was reported to impair the activation of the transcription factor Nrf2 following oxidative stress, to promote cell survival, to facilitate cell invasion and cell migration [60,61,62]. LA-triggered degradation of mutant p53, as observed in HT29 cells, might thus attenuate its pro-tumorigenic and metastatic activity. 

Furthermore, we show that LA-dependent depletion of p53 was restricted to the protein level, whereas gene expression of *p53* was only slightly affected. This finding is consistent with the notion that p53 is mainly regulated on the protein level by post-translational modifications, which can either stabilize p53 (phosphorylation, acetylation, etc.) or promote its degradation (ubiquitination) [53]. Depletion of p53 on protein level was also reported in lymphoblastoid cell lines deficient or proficient for the DDR kinase ATM, at a similar dose-dependent manner starting with 250 µM LA after 24 h [30]. Two other studies assessed the effects of LA on p53 phosphorylation on Ser-15, suggesting increased phosphorylation without changes in the total p53 protein level [31,32]. Ser-15 phosphorylation of p53 is known to be catalyzed by apical DDR kinases, such as ATM, following DNA damage [33,63]. However, no genotoxic effects of LA were observed in our previous study in HCT116 cells [15]. In contrast to the aforementioned studies and our own data, another work reported an accumulation of p53 in HCT116 cells after 24 h of incubation with 50 µM LA [19]. Unfortunately, the authors did not include dose–response experiments and were not able to detect a p53-dependent transcriptional response after LA exposure. 

The depletion of p53 in CRC cells occurred already after 24 h in a dose-dependent manner and preceded the cytotoxic effects of LA, which became visible in LS174T and HT29 cells after 48 h (Figure 2, Figure A1). The genotoxic anticancer drug 5-FU caused a p53-dependent transcriptional response with upregulation of *MDM2* and other p53-responsive genes, whereas this was not observed following LA treatment. The LA-mediated upregulation of *p21* at high doses is consistent with our previous study, which showed that p21 induction by LA is independent of p53 [15]. We further assessed cellular ROS formation and showed moderately increased ROS levels at 1000 µM LA, which is in agreement with the pro-oxidative effects of LA reported in several tumor cell lines [16,17,31]. However, pre-treatment of cells with the thiol-donor and antioxidant NAC did not prevent LA-induced degradation of p53, but rather promoted this process. Whether or not NAC affects LA-triggered cytotoxicity is an open question, which has to be addressed in futures studies. In contrast, the polyphenol curcumin with a well-known antioxidative activity [44] caused a moderate stabilization of p53 in HCT116 cells, which is consistent with a previous study [64]. These findings indicate that the disulphide and/or its reduced dithiol group are the structural elements required for p53 degradation.

Subsequently, we analyzed the underlying cellular process responsible for p53 degradation in more detail. The ubiquitin-proteasome system and autophagy are the two major quality control pathways to maintain cellular homeostasis [65]. As our previous study revealed autophagy induction by LA in HCT116 and RKO cells [13], we monitored autophagosome formation in all CRC cell lines tested. Consistent with our previous finding, increased autophagosome formation was detected in HCT116 and RKO cells, while only weak autophagy induction was observed in LS174T and SW48 cells (Figure 4). Nevertheless, the autophagy receptor p62 was strongly upregulated in all CRC cell lines. p62 is involved in selective autophagy via binding of ubiquitinated cargo molecules, which are then directed to the autophagosome for lysosomal degradation [66]. The expression of p62 is upregulated by the transcription factor Nrf2, which is activated upon oxidative stress [67]. Vice versa, p62 contributes to the activation of Nrf2 through inactivation of KEAP1, which targets Nrf2 for degradation under normal conditions [68], thereby creating a p62-Nrf2 feedback loop. Multiple lines of evidence show that LA causes activation of the Nrf2 pathway and upregulation of Nrf2-dependent gene expression [69,70,71]. The observed accumulation of p62 in CRC cells treated with LA could thus be attributable to the concomitant activation of Nrf2 by LA. Intriguingly, LA treatment caused a dose-dependent induction of Nrf2 and its downstream target HO-1 in CRC cells (Figure 5). Another reason for increased p62 levels may be its co-aggregation with accumulated cargo molecules as described previously [68]. However, neither pharmacological inhibition nor genetic abrogation of autophagy were able to rescue the LA-triggered depletion of p53 (Figure 4), hence excluding a major role for autophagy in p53 degradation. 

Apart from the abovementioned mutual regulation of Nrf2 and p62, Nrf2 activation can also increase proteolytic activity by upregulation of the 20S proteasome and the Pa28αβ (11 S) proteasome regulator, as demonstrated following oxidative stress and treatment with the Nrf2 inducers curcumin or LA [72]. Consistently, LA-dependent Nrf2 activation was observed in CRC cells, which coincided with p53 depletion. However, pharmacological inhibition of Nrf2 by ML385 was not able to rescue p53 degradation (Figure 5). It should be mentioned that the inhibitor itself had some effect on cell growth, indicating that a genetic approach may be more appropriate to elucidate the contribution of Nrf2 to the LA-triggered depletion of p53. 

In contrast, co-immunoprecipitation studies and western blot analysis revealed increased ubiquitination and proteasomal degradation of p53 by LA treatment, which can be blocked by proteasome inhibition (Figure 6). To figure out whether the E3 ubiquitin ligase MDM2 is responsible for the enhanced ubiquitination of p53, MDM2 levels were assessed on gene and protein level, showing no induction by LA. The pharmacological MDM2 inhibitor Nutlin-3a boosted p53 stabilization and the induction of the p53 targets p21 and MDM2, but did not abrogate the LA-mediated depletion of p53 and, concomitantly, p21 (Figure 7). These results indicate that MDM2 is dispensable for p53 degradation triggered by LA. Nonetheless, to completely exclude a contribution of MDM2, a genetic approach is required since MDM2 was reported to harbor two different binding sites for p53. The N-terminal p53 binding domain is targeted by Nutlin-3a, thereby restoring the transcriptional response by p53. However, Nutlin-3a hardly inhibits p53 ubiquitination [73], which is in line with the observed ubiquitinated p53 species in HCT116 cells after Nutlin-3a treatment (Figure 7). The ubiquitination of p53 by MDM2 is likely mediated by a second binding site in the central part of MDM2 [73]. Furthermore, it should be kept in mind that various other E3 ligases (e.g., Pirh2, COP1, Trim39, etc.) have been identified, which target p53 for ubiquitination and subsequent proteasomal degradation [74]. Thus, it is also conceivable that one of those E3 ligases may be modulated by LA.

The molecular trigger of this enhanced p53 degradation following LA exposure likely involves chemical modification of critical thiol groups (cysteine, methionine) within the p53 protein. The central DBD harbors 10 cysteine residues, of which 6 display surface orientation and are thus prone to undergo modification, e.g. by oxidants or electrophiles [26]. H_2_O_2_ was shown in vitro to oxidize the cysteine residues in the p53 core domain, which are responsible for zinc coordination [75]. Furthermore, oxidation of p53 destabilized p53 wild-type conformation and impaired its DNA binding in vitro, which could be reversed by the reducing agent DTT [76]. More specifically, oxidation of Met340 located in the tetramerization domain was reported to disrupt p53 folding [77]. 

Another possibility is the binding of LA as reactive disulphide to thiol-containing amino acids in p53, thereby destabilizing p53 conformation and facilitating its ubiquitination. Interestingly, the amino acids Cys182 and Cys277 present in the DBD of p53 are highly susceptible to chemical modification by the alkylating reagent *N*-ethylmaleimide [78]. In line with this finding, Cys residues in the proximal DBD including Cys182 were shown to be glutathionylated in tumor cells, which was further increased by anticancer drugs and oxidative stress [79]. Intriguingly, the drug disulfiram (DSF), which also contains a reactive disulphide bond, also catalyzed p53 glutathionylation and direct modification of p53. This resulted in the ubiquitin-proteasome mediated degradation of p53 in HCT116 and HT29 cells [80]. These findings are in striking conformance with our study, in which LA targeted p53 for enhanced degradation by the ubiquitin-proteasome machinery. 

Finally, we demonstrate that LA attenuated the p53-dependent induction of the cell cycle regulator p21 after treatment with the genotoxic anticancer drugs 5-FU and doxorubicin. The combination regimen of LA and the anticancer drugs led to synergistic killing of CRC cells in a p53-dependent manner as determined by Chou–Talalay analysis (Figure 8, Table 2). Strikingly, this synergism disappeared when p53-deficient HCT116 cells were used. It is yet conceivable that also proteins other than p53 and p21 may contribute to the observed synergism. These results extend our previous study, which showed that LA potentiates the cytotoxicity of both 5-FU and the temozolomide in CRC cells [13,15]. It is further remarkable that the combination of LA and doxorubicin provided the best synergistic effect both in viability studies and cell death measurements in CRC cells with p53 expression. In support of the data presented herein, LA increased apoptotic cell death induction by etoposide and ionizing radiation [19]. LA was further reported to enhance the cytotoxicity of the antineoplastic drugs cis-Platin and etoposide in lung cancer cells [81]. This body of evidence suggests that LA and its chemical derivatives [9] are promising agents for targeting cancer cells. 

In conclusion, our study showed for the first time that LA triggers the degradation of both wild-type and mutant p53 in a ubiquitin-proteasome dependent manner and elicits synergistic cell killing with genotoxic anticancer drugs in CRC cells by p53 degradation.

## Figures and Tables

**Table 1:** Primers used for qPCR

qPCR Primer	Sequence (5′–3′)
***ACTB***-real-up	TGGCATCCACGAAACTACC
***ACTB***-real-low	GTGTTGGCGTACAGGTCTT
***FASR***-real-up	TTATCTGATGTTGACTTGAGTAA
***FASR***-real-low	GGCTTCATTGACACCATT
***GADD45a***-real-up	ATCTCCCTGAACGGTGAT
***GADD45a***-real-low	TGTAATCCTTGCATCAGTGT
***GAPDH***-real-up	CATGAGAAGTATGACAACAG
***GAPDH***-real-low	ATGAGTCCTTCCACGAT
***MDM2***-real-up	ATCTTGATGCTGGTGTAA
***MDM2***-real-low	AGGCTATAATCTTCTGAGTC
***NOXA***-real-up	TCTTCGGTCACTACACAAC
***NOXA***-real-low	CCAACAGGAACACATTGAAT
***p21***-real-up	ACCATGTCAGAACCGGCTGGG
***p21***-real-low	TGGGCGGATTAGGGCTTC
***PUMA***-real-up	TAAGGATGGAAAGTGTAG
***PUMA***-real-low	TTCAGTTTCTCATTGTTAC
***p53***-real-up	AGCACTAAGCGAGCACTG
***p53***-real-low	ACGGATCTGAAGGGTGAAA

**Table 2:** Combination indexes (CI) determined by ATP assays and the Chou–Talalay-method

Cell Line/CI	LA + 5-FU	LA + Doxo
HCT116	0.67	0.55
HCT116-p53^−/−^	1.22	1.29
RKO	0.84	0.59

## Abbreviations

5-FU	5-fluorouracil
ARE	antioxidant response element
CDT	cytolethal distending toxin
CI	combination index
CRC	colorectal cancer
CQ	chloroquine
DBD	DNA binding domain
DDR	DNA damage response
DHLA	dihydrolipoic acid
Doxo	doxorubicin
DSF	disulfiram
HO-1	heme oxygenase 1
LA	ɑ-lipoic acid
MDM2	mouse double minute 2 homolog
MGMT	*O*^6^-methylguanine-DNA methyltransferase
NAC	N-Acetyl-cysteine
NEM	*N*-ethylmaleimide
NRF2	nuclear factor erythroid 2-related factor 2
PI	propidium iodide
ROS	reactive oxygen species
RT	room temperature
scr	scrambled
SEM	standard error of the means
